# Michigan State Board of Health

**Published:** 1885-08

**Authors:** 


### MICHIGAN STATE BOARD OF HEALTH.

(Reported for the Chicago Medical Journal and Examiner.)

At the quarterly meeting of the State Board of Health of Michigan, held July 14, 1885, at its office in Lansing, the following members were present: Drs. Avery, Lyster, Hazlewood, Vaughan, Tyler and Baker.

The board spent much time in examining plans, as required by law, of a proposed new double cottage at the Reform School at Lansing, a building designed to accommodate 100 pupils. The plans were submitted by the architect, Mr. Wm. Appleyard, of Lansing. The board approved the plans for ventilating and warming the second and third floors. With reference to the first floor, the board recommended, in order to avoid the settling of dust in the registers, that iron hoods be placed over the fresh-air inlets which enter the school-rooms through the floor, and that two additional foul-air outlets, each of a size equal to that of the present outlet, be provided for the two school-rooms,—one in the west wall of the west room, and the other in the east wall of the east room, and that these shafts be heated by steam pipes. Each of these two school-rooms is thirty by forty feet, and ten feet high, designed to accommodate fifty pupils. Fresh air is brought into each room by two shafts, having an area of 512 square inches. The foul-air shaft for each room is only one-half the size of the inlet pipes; the area being 256 square inches. The recommendation by the Board of Health is that the outlets equal the inlets in area. The method of heating and of taking fresh air into the building was approved, and also the making of the basement floor of cement troweled smooth.

The secretary presented a printed statement in regard to the charges made by a minority of the legislative Committee on Investigation, and asked that a committee be appointed to fully investigate the conduct of the office, and the several laws governing the action of this board. Drs. Hazlewood, Vaughan and Tyler were made such a committee.

The Secretary read a *résumé* of the work of other state Boards of Health, and of city boards of health; also a report in regard to recent legislation in Michigan in regard to subjects relating to public health. The act passed by the legislature appropriating ten thousand dollars as an epidemic contingent fund was also read by the secretary, and the board appointed Drs. Baker and Lyster a committee to frame rules under that law, and submit them to the members, so that they can be in readiness in case of threatened occurrence of cholera, small-pox, etc. The board authorized the secretary, should cholera appear in Michigan, immediately to proceed to the place, or send a competent person to confer with and aid the local board in restricting the spread of the disease; and it was informally agreed that if cholera appeared in the United States, the president of the board should at once call a meeting. The revision of the document on restriction of cholera, issued by the board in 1884, and the printing of 20,000 copies for distribution, were ordered.

The Board directed that the names and addresses of health officers in Michigan be printed in pamphlet form.

The subject of typhoid fever was discussed, and the secretary was instructed to issue a circular designed to collect information from health officers in localities where typhoid fever occurs; and a committee was appointed to prepare and have printed a document giving methods of preventing and restricting typhoid fever, the document to be distributed in localities where it occurs.

The board considered the subject of the carrying of infected articles and bodies, dead from contagious diseases, and circulars relating to that subject were ordered to be printed and sent to health officers and to railroad companies.

The following is from the secretary's report of work done during the quarter ending, July 13, 1885, the leading features of which are as follows: The weekly and monthly bulletins of health in Michigan, and the meteorology and mortality reports had been prepared from the numerous reports received, and sent out as heretofore. The footings and computations on meteorological registers and on the sickness reports and tables have been carried on; and the meteorological computations for the year 1884 nearly completed ready for tabulation. The office had made large distributions of documents relative to the work of health officers, and to the restriction of contagious diseases, to newly appointed health officers, and to others, especially in localities where such diseases have occurred. The proceedings of the sanitary convention at Lansing have been edited, sent to the printer, and the proof on most of it read. Articles on meteorology and sickness in Michigan in 1883 have been completed from data previously collected. Data collected by the office relative to scarlet fever in Michigan in 1884 have been compiled; and also those relative to diphtheria. A map has been prepared showing the distribution of diphtheria in Michigan in 1884. Small-pox has been present in Michigan during the quarter: at Bellevue, Eaton county; Alba, Antrim county; Battle Creek, Girard township; Branch county, and South Haven. The outbreak at South Haven was confined to those first exposed, and has been stampted out, after nine cases occured with one death. The infection at South Haven was from a German immigrant who sailed from Bremen, April 12th, on the ship *Donan*,

North German Lloyd line. The immigrant was broken out with small-pox when he reached South Haven, April 27, and might have been quarantined *en route*, and the outbreak thus confined to the one case. All infected persons were at once vaccinated by the health officer, but the virus was not good, and thus precious time was lost. This outbreak is but another added to the many constantly recurring outbreaks of communicable diseases in Michigan and the northwest, to which a faithfully executed immigrant inspection service, carried on by the national government, would put an end, or greatly lessen. At the present, so far as known, there is not a case of small-pox in Michigan. Typhus fever was reported at Grand Rapids, during the week ending July 4th.

Cholera is spreading with great violence in Mediterranean Spain, hundreds are dying daily. It was reported as present in Marseilles over a month ago, and July 10th, at Toulon. A strange and fatal disease, believed to be cholera, was also reported from Portugal. Asiatic cholera will probably reach this country this year or next year, and the State Board of Health has prepared to meet the emergency by many lines of work, as best it could. About twelve thousand copies of the document on the best method for the prevention and restriction of cholera were distributed to the people last year. The recent distribution of documents relative to typhoid fever, and especially the correspondence with health officers throughout many parts of the state, on the best method of restricting this disease, has done something in the way of drill in the two important methods applicable in case of cholera,—the disinfection of all bowel discharges and the protection of the purity of the water supply. Much, however, remains to be done in many localities in the way of abating nuisances, and in protecting wells from sources of contamination. The legislature has passed an act granting to the State Board of Health power to establish a system of inspection of immigrants and travelers, and the disinfection of baggage, etc., liable to be infected with cholera or other dangerous communicable disease ; but the act was not given immediate effect and so does not take effect until September 18th, 1885. The contingent appropriation to enable the Board to carry on the inspection, etc., provided for in the act can be used on or after September 18th, in case the Governor thinks its use is necessary. Reports relative to examinations during this quarter, by this board, of plans for buildings, have been sent to the boards governing the Michigan Asylum for the Insane at Kalamazoo, the Northern Asylum for the Insane at Traverse City, and the State Reform School at Lansing. Over six hundred and fifty pages of the letter-book have been used in copying the most important parts of the correspondence, and other branches of the office work has been large during the quarter, and the legislature made additional demands upon the time of the office.

A very successful Sanitary Convention was held during the quarter at Ypsilanti.

